# Relationship between the corticostriatal terminals from areas 9 and 46, and those from area 8A, dorsal and rostral premotor cortex and area 24c: an anatomical substrate for cognition to action

**DOI:** 10.1111/j.1460-9568.2007.05825.x

**Published:** 2007-10

**Authors:** Roberta Calzavara, Philippe Mailly, Suzanne N Haber

**Affiliations:** 1 Department of Pharmacology and Physiology, University of Rochester School of Medicine 601 Elmwood Avenue, Rochester, New York 14642, USA; 2 Laboratory Neurobiologie des Signaux Intercellulaires, University, Pierre & Marie Curie UMR 7101, CNRS, 7 quai St Bernard, 75252, Paris, France

**Keywords:** action control, anatomy of the basal ganglia, executive function, macaque monkey, premotor cortex

## Abstract

Our previous data indicate that there are specific features of the corticostriatal pathways from the prefrontal cortex. First, corticostriatal pathways are composed of focal, circumscribed projections and of diffuse, widespread projections. Second, there is some convergence between terminal fields from different functional regions of the prefrontal cortex. Third, anterior cingulate projections from area 24b occupy a large region of the rostral striatum. The goal of this study was to determine whether these features are also common to the corticostriatal projections from area 8A (including the frontal eye field; FEF), the supplementary eye field (SEF), dorsal and rostral premotor cortex (PMdr) and area 24c. Using a new approach of three-dimensional reconstruction of the corticostriatal pathways, along with dual cortical tracer injections, we mapped the corticostriatal terminal fields from areas 9 and 46, 8A-FEF, SEF, PMdr and 24b and c. In addition, we placed injections of retrogradely transported tracers into key striatal regions. The results demonstrated that: (i) a diffuse projection system is a common feature of the corticostriatal projections from different frontal regions; (ii) key striatal regions receive convergent projections from areas 9 and 46 and from areas 8A-FEF, SEF, PMdr and 24c, suggesting a potential pivotal role of these striatal regions in integrating cortical information; (iii) projections from area 24c, like those from area 24b, terminate widely throughout the striatum, interfacing with terminals from several frontal areas. These features of the corticostriatal frontal pathways suggest a potential integrative striatal network for learning.

## Introduction

Learning and planning an action require complex interactions between frontal cortex and the dorsal striatum. Although unique functions are difficult to assign to specific frontal regions, connectivity studies demonstrate general relationships between frontal cortical areas. Areas 9 and 46 are primarily connected to other regions of prefrontal cortex, both within areas 9 and 46 and with areas 8A (including the frontal eye field; FEF), 32 and 24b (Barbas, 1992; Carmichael & Price, 1996; Petrides & Pandya, 1999). In addition, there are weaker projections to the dorsal and rostral premotor area (PMdr), the supplementary eye field (SEF) and the rostral motor cingulate cortex (area 24c; Barbas & Pandya, 1987; Petrides & Pandya, 1999). In contrast, PMdr is primarily connected to other premotor areas and with area 24c (Petrides & Pandya, 1999; Luppino *et al*., 2003; Takada *et al*., 2004). In addition, there are weaker connections with areas 9 and 46 (Barbas & Pandya, 1987; Luppino *et al*., 2003).

Integration of diverse functional information has been extensively discussed for corticocortical networks (Paus, 2001; Rowe *et al*., 2005). However, corticostriatal projections are considered to be organized in parallel segregated circuits (Middleton & Strick, 2002). We recently identified three features of the prefrontal corticostriatal projections which indicate a more complex network, providing an anatomical substrate for potential integration of information across functional circuits (Haber *et al*., 2006). First, there are two corticostriatal projection patterns from each prefrontal region: a focal projection field forming the well described, relatively confined, terminal patches (Graybiel & Penney, 1999), and a diffuse projection that consists of fibres that innervate a broad striatal region cutting across functional domains. Second, there is convergence in the rostral striatum between focal projections from areas 9 and 46, 24b and orbital frontal cortex (OFC). Finally, unlike other prefrontal regions, the terminal field from area 24b extends broadly throughout the rostral striatum.

The goal of this study was to investigate whether these features are also common to more caudal frontal cortex, areas 8A-FEF, SEF, PMdr and 24c, indicating a general rule for the frontal corticostriatal pathways. Moreover, we wanted to determine differences in the pattern of corticostriatal projections from areas 9 and 46, and the potential interaction between these projections and those from caudal frontal areas. We compared patterns of corticostriatal projections from areas 8A-FEF, SEF, PMdr and 24c with those from areas 9, 46 and 24b to determine: (i) whether areas 8A-FEF, SEF, PMdr and 24c have a diffuse projection (like areas 9, 46 and 24b) to the striatum, in addition to their focal projection; (ii) whether there is convergence of focal projections from areas 9, 46 and 24b with those from areas 8A-FEF, SEF, PMdr and 24c; (iii) whether area 24c has a widespread projection (like area 24b), indicating a general feature of the dorsal anterior cingulate cortex (dACC).

## Materials and methods

To analyse the distribution patterns of the corticostriatal projections, we injected anterograde and bi-directional tracers into areas 9 and 46, into a subregion of area 8A including the FEF (Parthasarathy *et al*., 1992), into the SEF (Schlag & Schlag-Rey, 1987), into PMdr and into the dACC (specifically areas 24b and 24c) of the macaque monkey. The entire projection field was charted throughout the striatum for each case. Corticocortical labelling was used to verify the specificity of the injection sites. In addition to the traditional charting of individual fibres, the densest projection fields, referred to as focal projections, were outlined for each case to create three-dimensional (3-D) maps of these fields (see pages 10–11). The 3-D maps of the focal projections were then compiled to delineate the striatal regions that received primary input from these frontal areas. We used the 3-D reconstructions to determine potential relationships between the focal terminal fields from each cortical functional region. Moreover, to illustrate the extent of the entire terminal field from each cortical functional region we compiled two-dimensional (2-D) charts of the fibre clusters (or diffuse projections) outside the focal projection field, along with 2-D charts of focal projections, in one map. The maps of the diffuse and focal projections from different functional regions were then joined together to determine the extent of potential interaction between different corticostriatal pathways. To verify the potential relationships between the terminal fields from different cortical regions we carried out: (i) experiments involving multiple anterogradely transported tracer injections placed into different frontal areas; and (ii) experiments involving multiple retrogradely transported tracer injections placed into selected areas of the striatum. Additionally, for this purpose we also referred to previous data from injections of retrogradely transported tracers into the dorsal striatum (Haber *et al*., 2000; McFarland & Haber, 2000).

### Surgery and tissue preparation

Twenty adult macaque monkeys (15 *Macaca nemestrina*, four *Macaca fascicularis* and one *Macaca mulatta*) were used for the tracing studies. All the experimental procedures and care of laboratory animals conformed to, and were approved by, the University Committee on Animal Resources (UCAR) and the ILAR Guide for the Care and Use of Laboratory Animals (Institute of Laboratory Animal Resources CoLS, National Research Council, 1996).

The surgical procedures were performed under sterile conditions. Animals received initial anaesthesia with Ketamine (10 mg/kg, intramuscularly) and a surgical level was maintained via isoflurane. In a subset of cases in which physiological recording was necessary to locate the injection sites in the striatum (injections of retrogradely transported tracers), pentobarbital (initial dose of 20 mg/kg, intravenously, and maintained as needed) was used to maintain a deep anaesthesia. During surgery, temperature, heart rate and respiration were monitored and hydration was maintained with saline (intravenously). Animals were placed in a David Kopf Instruments stereotaxic apparatus (Tujunga, CA, USA), a midline scalp incision was made and the muscles and fascia were displaced laterally to expose the skull. A craniotomy (∼ 2–3 cm^2^) was made over the region of interest and small dural incisions were made only at the injection sites. In several animals, we obtained magnetic resonance images (MRI; 3 Tesla) in order to guide our injection sites. In the absence of MRI images, the cortical injection sites were chosen by visual inspection of anatomical landmarks such as the cortical fissures. To guide the deep injections in the striatum, serial electrode penetrations were made throughout the rostrocaudal and mediolateral extents of the striatum to identify neuronal activity based on patterns of electrophysiological recordings (Haber *et al*., 1993). The location of neurons encountered was used to map the boundaries of different basal ganglia structures. The absence of cellular activity signaled an area of fibre tracts, i.e. the corpus callosum, the internal capsule and the anterior commissure. Accurate placement of tracer injections was achieved by careful alignment of the injection cannulas with the electrode.

Animals received an injection of one or more of the following anterograde or bi-directional tracers: Lucifer Yellow (LY), Fluororuby (FR) or Fluorescein (FS) conjugated to dextran amine [40–50 nL, 10% in 0.1 m phosphate buffer (PB), pH 7.4; Invitrogen, Carlsbad, CA USA]; *Phaseolus vulgaris*–leucoagglutinin (PHA-L; 50 nL, 2.5%; Vector Laboratories, Burlingame, CA, USA); or tritiated amino acids (AA; 100 nL, 1 : 1 solution of [^3^H] leucine and [^3^H] proline in dH_2_O, 200 mCi/mL; NEN, Boston, MA, USA). Tracers were pressure-injected over 10 min using a 0.5-µL Hamilton syringe. Following each injection, the syringe remained *in situ* for 10–20 min. Upon recovery from anaesthesia, the animals were returned to their home cages and closely monitored.

Twelve to fourteen days post-surgery, animals were initially anaesthetized with Ketamine (10 mg/kg, intramuscularly) and then deeply anaesthetized with pentobarbital (25 mg/kg, intravenously) and transcardially perfused with saline followed by a 4% paraformaldehyde and 1.5% sucrose solution in 0.1 m PB, pH 7.4. Brains were postfixed overnight and cryoprotected in increasing gradients of sucrose (10, 20 and 30%). Serial sections of 50 µm were cut on a freezing microtome into cryoprotective solution as previously described (Haber *et al*., 1993).

Immunohistochemistry was performed on free-floating sections (one in eight for each tracer; 400-µm interval) to visualize LY, FR, FS and PHA-L labelling of cells and fibres. Prior to incubation in primary antiserum, tissue was treated with 10% methanol and 3% hydrogen peroxide in 0.1 m PB to inhibit endogenous peroxidase activity, rinsed for 1–2 h in 0.1 m PB with 0.3% Triton X-100 (TX; Sigma, St Louis, MO, USA) and preincubated in 10% normal goat serum (NGS) and 0.3% TX in PB for 30 min. Tissue was then placed in the primary anti-LY (1 : 3000 dilution; Invitrogen), anti-FS (1 : 2000; Invitrogen), anti-FR (1 : 3000; Invitrogen) or anti-PHA-L (1 : 1000; EY Laboratories, San Mateo, CA, USA) in 10% NGS and 0.3% TX in 0.1 m PB for four nights at 4 °C. Following extensive rinsing, the tissue was preincubated in 10% NGS and 0.3% TX in PB for 30 min and then incubated in biotinylated secondary antibody followed by incubation with the avidin–biotin complex solution (Vectastain ABC kit; Vector Laboratories). Immunoreactivity was visualized using standard 3,3′-diaminobenzidine tetrahydrochloride (DAB) procedures. Staining was intensified by incubating the tissue for 1–15 min in a solution of 0.05% DAB, 0.025% cobalt chloride, 0.02% nickel ammonium sulphate and 0.01% H_2_O_2_ to yield a black reaction product. Sections were mounted onto gel-coated slides, dehydrated, defatted in xylenes and coverslipped with Permount. In the dual cortical injection cases, adjacent sections were processed for each antibody reaction to avoid crossreactivity. Adjacent sections (one in eight) were stained for Nissl.

### Data analysis

**Fig. 7:**
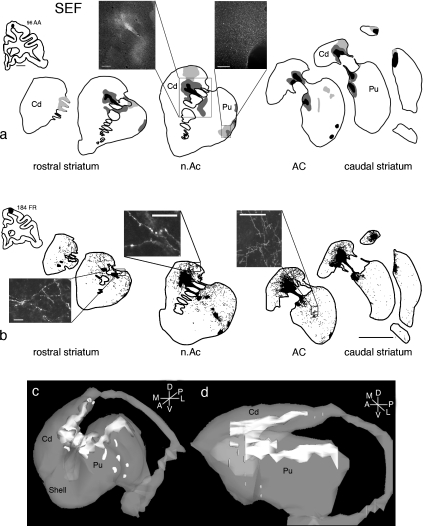
Projections from the SEF to the striatum. (a and b) Schematic chartings and microphotographs of the terminals following two injections into the SEF: (a) case 96, tritiated AA; (b) case 184, FR. The focal projection fields (black solid areas) and the diffuse projection fields are shown in charts at different AP levels: rostral striatum, AP 28–26; n. Ac, AP 22; AC, AP 18.5; and caudal striatum, AP 14–10. The diffuse terminal field for the tritiated AA injection (a, case 96) is shown as a shaded area in grey surrounding the focal projections. Microphotographs at low and high magnifications illustrate the focal and diffuse terminal fields. (c and d) 3-D rendering of the focal collective projections from the SEF shown in (c) coronal and (d) lateral views of the striatum. Scale bars, 5 mm (a and b schematics); microphotographs: 1 mm (lowest power); 200 µm (a) and 100 µm (b), (intermediate power); and 25 µm (highest power).

Using counterstained or adjacent Nissl-stained coronal sections, we determined the boundaries of cytoarchitectonic areas for each injection site according to previous anatomical classifications (Walker, 1940; Matelli *et al*., 1985, 1991; Paxinos *et al*., 2000). Cortical injections with contamination or weak labelling were eliminated from the analysis. Contamination refers to all injections in which the tracer was not limited to a single cortical region but had leaked into an adjacent area or into the underlying white matter. Weak labelling refers to relatively few labelled fibres in the striatum, typically the result of the injection site centred in superficial cortical layers. We charted all thin, labelled corticostriatal fibres showing clear boutons. Thick fibres without clear terminal boutons were assumed to be passing fibres and were not included. Fibre distributions for each case were charted throughout the entire rostrocaudal extent of the striatum. In cases with tritiated AA injections, the charting of the entire diffuse projection field was limited by the inability to detect discrete fibre morphology. In these cases, denser silver grain labelling over background (observed with the 10 or 20× objectives of the light microscope) determined striatal areas of the diffuse projection. The pattern of diffuse projection following one tritiated AA injection (case 96; Fig. 7a) was confirmed by a second FR injection in the same cortical region (case 184; Fig. 7b). For the injections of retrogradely transported tracers into the striatum, we charted all labelled cells in the cortex.

There were 22 injections into different areas of the frontal cortex. The SEF is located in the rostral and dorsomedial aspect of the premotor cortex (area 6). However, given the oculomotor properties of the SEF, in this paper we describe it as a region that is functionally distinct from PMdr and therefore when we refer to PMdr we are excluding the SEF. There were seven injections into area 9, three in area 46, one into area 8A-FEF, two into the SEF, four into PMdr and five into the rostral cingulate cortex (three into area 24b and two into area 24c). To test the potential convergence between terminals from different frontal areas, three monkeys (cases 161, 166 and 184) received dual cortical injections, one into areas 9 and 46 and one into PMdr (cases 161 and 166) or SEF (case 184). In all cases, the general corticocortical labelling was consistent with expected results from previous studies (Petrides & Pandya, 1999; Barbas & Pandya, 1987; Huerta *et al*., 1987; Kurata, 1991; Lu *et al*., 1994; Carmichael & Price, 1996; Luppino *et al*., 2003; Takada *et al*., 2004). In addition, to further test the potential convergence between focal projections from different frontal areas in key striatal regions, we placed injections of retrogradely transported tracers into two striatal regions of convergence that were predicted by the 3-D model.

### 3-D reconstructions

**Fig. 1:**
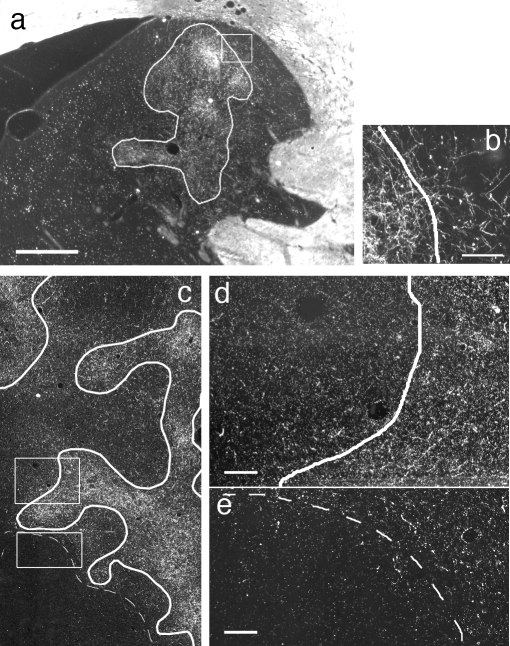
Microphotographs at low and high magnifications of the corticostriatal projections following tracer injections into (a and b) PMdr (case 161, FR) and (c–e) area 24c (case 62, tritiated AA). In (a) and (c), the charted outlines of the focal projections have been superimposed on the microphotographs. In (b) and (d), regions around the borders of the focal projections are shown at higher magnification. In (e), a region around the border of the diffuse projection vs. background (case 62, tritiated AA injection) is shown at higher magnification. Scale bars, 1 mm (a and c), 100 µm (b, d and e).

3-D reconstructions of focal projection fields in the striatum were developed to: (i) address how each focal projection lies within the striatum in dorsal–ventral, medial–lateral and anterior–posterior space; and (ii) understand the relationships and predict convergence between focal projections from different cortical regions (Haber *et al*., 2006). Previous studies have shown (at low magnification) relatively large, dense patches of corticostriatal projections (Yeterian & Van Hoesen, 1978; Selemon & Goldman-Rakic, 1985; Parthasarathy *et al*., 1992). We used the ability to visualize these dense projections at low magnification to create the 3-D renderings for the focal projections. Therefore, the focal projections were determined at the light microscope using a 1.6× objective and directly outlined using the Neurolucida software (MicroBrightField, Inc.). Adjacent patches of dense fibres (visible at 1.6×) were often surrounded by less dense fibre labelling (visible at 4×). These were included within the outline of the focal projection. Isolated patches (visible at 1.6×) were charted as individual outlines. Boundaries for each focal projection were checked for accuracy with the chartings that were carried out at higher magnification (10×; Fig. 1). The outlines of the focal projections, generated with the aid of the Neurolucida software, were exported into the software for 3-D reconstruction (IMOD software; Boulder laboratory for 3-D Electron Microscopy; Kremer *et al*., 1996) and a 3-D reconstruction that contained the focal projection field was created for each case separately.

To merge several cases together, we developed a reference model of the striatum from one animal. Data from each case were then transposed into the reference striatum using landmarks of key internal structures surrounding the striatum. Following the transposition of focal projections from each case, every contour placed in the reference model was checked with the original for medial–lateral, dorsal–ventral and anterior–posterior placement, and relative size. This ensured that the focal projection field from each case was accurately placed with respect to its position and the proportion of the striatum it occupied. Thus, a 3-D rendering was created first for each single case and then for the combination of cases (for details, see Haber *et al*., 2006). To predict convergence between focal projection fields, the model was ‘sectioned’ throughout its rostrocaudal extent. Verification of convergence was tested with dual cortical injections into a single animal and with injections of retrogradely transported tracers into the striatum.

For illustrative purposes, we used four representative anterior–posterior levels, corresponding to the rostral pole (AP, ∼ 28–26), nucleus (n.) accumbens (AP, ∼ 22), anterior commissure (AP, ∼ 18.5) and caudal striatum (AP, ∼ 14–10), to describe the corticostriatal terminal fields.

## Results

Projections to the striatum from areas 9 and 46, 8A-FEF, SEF, PMdr and 24b and c extended from the rostral pole to the posterior regions of the caudate n. and putamen. However, the striatal area rostral to the anterior commissure received the densest overall innervation from these cortical regions. Consistent with our previous results (Haber *et al*., 2006), following all injections there were two components of the corticostriatal projections, focal and diffuse. The focal projections were comprised of dense terminals and showed a general topographic organization. In contrast, the diffuse projections, comprised of smaller clusters of terminals and fibres, were distributed widely throughout the striatum. The present data showed that a diffuse system is a common feature of the corticostriatal projections from areas 9 and 46, 8A-FEF, SEF, PMdr and 24b and c. Moreover, the collective data (from the 3-D reconstructions of the focal corticostriatal projections, dual cortical injections and injections of retrogradely transported tracers into the striatum) demonstrated both convergence and interdigitation between corticostriatal projections from different frontal areas. Finally, projections from areas 24b and c terminated widely throughout the striatum, interfacing with terminals from several frontal areas.

### Corticostriatal projections: focal and diffuse components of the terminal field

#### Areas 9 and 46

**Fig. 2:**
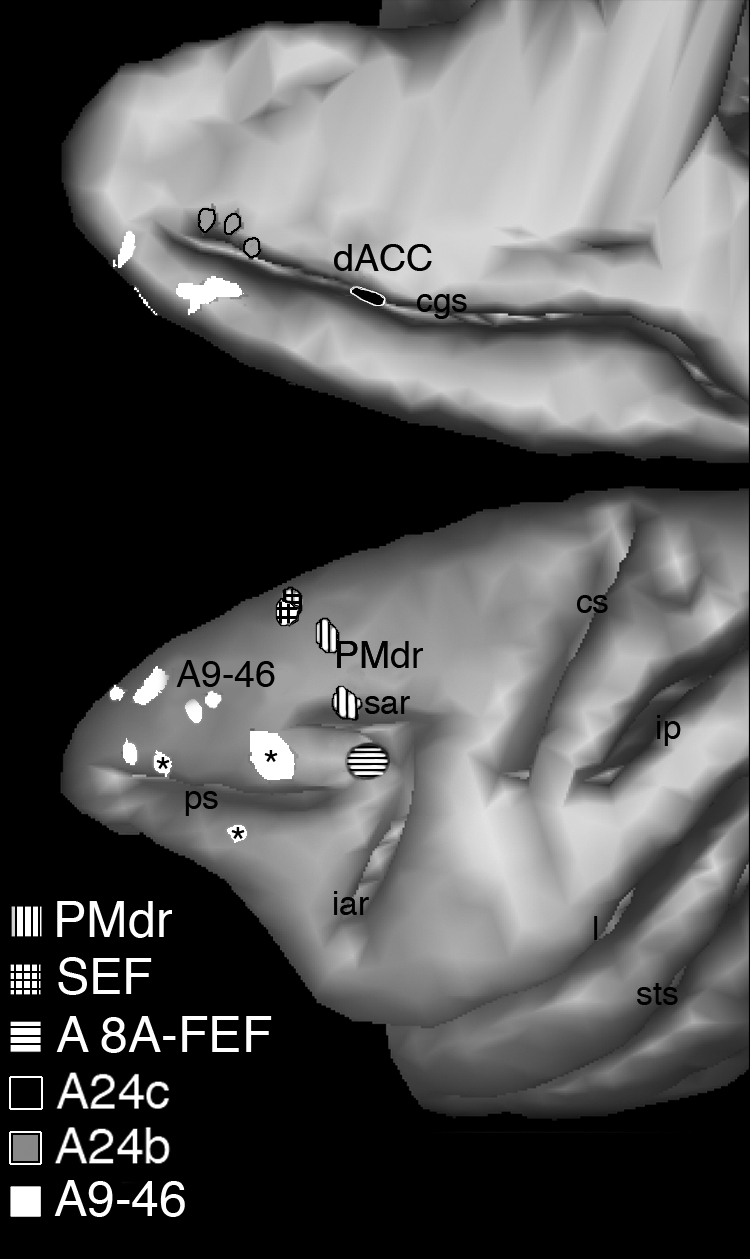
Three-dimensional reconstruction of the medial and lateral views of the brain, depicting the injection sites. Note: two adjacent injection sites in area 9 are represented as one large injection site, and two adjacent injection sites in SEF also appear as one large injection site. White, areas 9 and 46 (A9–46; the asterisks indicate injections in area 46); grey with black border, area 24b; black with white border, area 24c; black and white horizontal stripes, area 8A-FEF; black and white horizontal and vertical stripes, SEF; black and white vertical stripes, PMdr. Note that injection sites collectively cover a limited portion of each functional region. Abbreviations in this and subsequent Figs are in the main list.

There were three injections into medial area 9 and four into lateral area 9 (Fig. 2). Following the injections into medial area 9 there was dense labelling in areas 24b and 32 but not 24c, whereas there was weaker labelling in the SEF and no labelling in area 46 or area 8A-FEF. In contrast, the injections into lateral area 9 resulted in corticocortical labelling in both rostral and caudal area 46 and in area 8A-FEF. However, similar to medial 9, lateral area 9 had connections with areas 32 and 24b but not 24c. In addition, there were weaker connections with the SEF and PMdr. There were two injections into rostral area 46 and one into caudal area 46 (Fig. 2). The rostral injections resulted in corticocortical labelling in both medial and lateral areas 9, in caudal area 46 and in area 8A-FEF. In addition, there were weaker connections with the SEF, PMdr and area 32 but not with areas 24b or c. The caudal injection resulted in cortical labelling in area 9 (primarily the lateral part), in rostral area 46 and in area 8A-FEF. In addition, there were weaker connections with the SEF, PMdr and area 24c, but not with areas 24b or 32.

#### Focal projections.

**Fig. 3:**
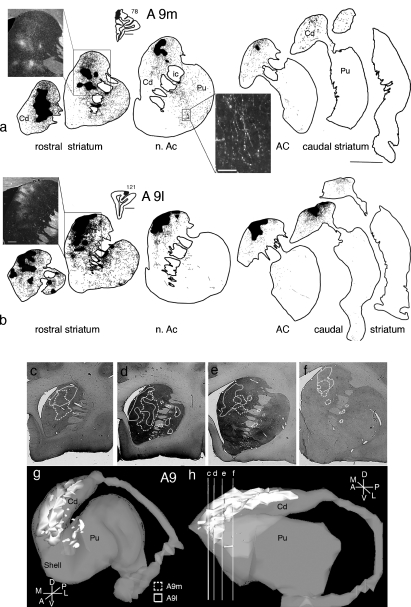
Projections from area 9 to the striatum. (a and b) Schematic chartings and microphotographs of the terminals following injections into the medial (a, case 78) and lateral (b, case 121) area 9. The focal projection fields (black solid areas) and the diffuse projection fields are shown in charts at different AP levels: rostral striatum, AP 28–26; n. Ac, AP 22; anterior commissure (AC; AP 18.5) and caudal striatum (AP 14–10). Cd, caudate n.; ic, internal capsule; Pu, putamen. Microphotographs at low and high magnifications illustrate the focal and diffuse terminal fields. (c–f) Coronal slices through the 3-D model with the corresponding Nissl section. Focal projections from medial and lateral area 9 are indicated with dotted and continuous lines, respectively. (g and h) 3-D rendering of the focal collective projections from area 9 shown in (g) coronal and (h) lateral views of the striatum. White lines in (h) indicate the level of sections illustrated in (c) – (f) Scale bars, 5 mm (a and b), 1 mm and 50 µm (low- and high-power photomicrographs, respectively).

**Fig 4:**
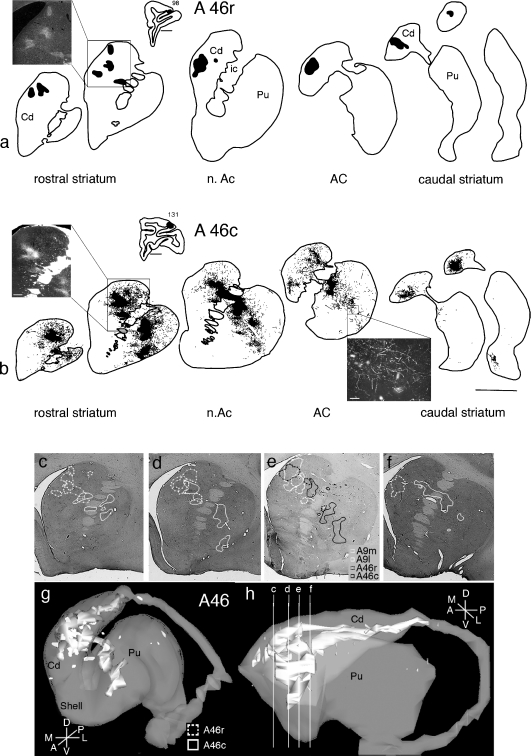
Projections from area 46 to the striatum. (a and b) Schematic chartings and microphotographs of the terminals following injections into the (b) rostral (case 98) and (b) caudal (case 131) area 46. The focal projection fields (black solid areas) and the diffuse projection fields are shown in charts at different AP levels: rostral striatum (AP 28–26), n. Ac (AP 22), AC (AP 18.5) and caudal striatum (AP 14–10). The diffuse terminal field for the tritiated AA injection (a, case 98) is shown as shaded areas in grey surrounding the focal projections. Microphotographs at low and high magnification illustrate the focal and diffuse terminal fields. (c–f) Coronal slices through the 3-D model with the corresponding Nissl section. Focal projections from rostral and caudal area 46 are indicated with dotted and continuous lines, respectively. (e) Summary section illustrating the topographic organization of the focal projections from different parts of areas 9 and 46 (white dotted line, medial area 9; white continuous line, lateral area 9; black dotted line, rostral area 46; black continuous line, caudal area 46). (g and h) 3-D rendering of the focal collective projections from area 46 shown in (g) coronal and (h) lateral views of the striatum. White lines in (h) indicate the levels of sections illustrated in (c) – (f) Scale bars, 5 mm (a and b), 1 mm and 100 µm (low- and high-power photomicrographs, respectively).

There was a complex topography of focal projections from different regions of areas 9 and 46. Area 9 projected primarily to the caudate n. (Fig. 3), whereas projections from area 46 also terminated in the putamen and continued caudal to those from area 9 (Fig. 4). As the fibres from areas 9 and 46 entered the striatum, they occupied a relatively large proportion of the rostral pole. Here, focal projections from medial area 9 terminated in the central part of the caudate n., while those from the lateral area 9 terminated in the medial and dorsal caudate n. (Fig. 3a and b, and c–f, respectively). At these rostral levels, focal projections from rostral area 46 terminated in the central and medial caudate n. (Fig. 4a) ventral to, and partially converging with, those from area 9 (Fig. 4e). In contrast, focal projections from caudal area 46 terminated primarily in the lateral caudate n., adjacent to the internal capsule, extending in the putamen (Fig. 4b–f). Caudal to the anterior commissure level, the collective focal projections from areas 9 and 46 primarily occupied the same region of the caudate n. and were no longer found in the putamen (Figs 3 and 4).

#### Diffuse projections.

Although the focal projection from areas 9 and 46 occupied the dorsal caudate n. and parts of the putamen, clusters of fibres from these areas extended into the rostral and ventral striatum, as previously reported (Haber *et al*., 2006). Diffuse labelling, particularly following injections into area 9, was also prominent in the dorsal and lateral striatum, rostral to the anterior commissure (Figs 3a and b, and 4b). Posterior to the anterior commissure level, diffuse projection continued to invade much of the caudate n. and some fibres were also found in the caudal and ventral putamen particularly following injections into area 46 (Figs 3a and b, and 4b).

**Fig. 5:**
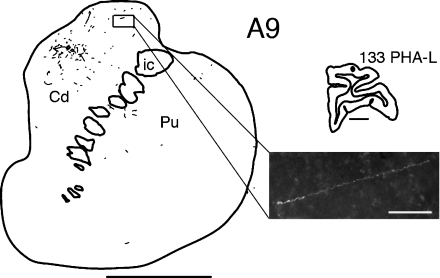
Corticostriatal projections following a small PHA-L injection into area 9 (case 133) are illustrated in a chart of the striatum at the n. Ac level. Microphotograph at high magnification illustrates an isolated labelled fibre in the lateral Cd n. Note that the pattern of focal vs. diffuse projections is also distinguishable following a confined injection site. Scale bar, 5 mm; microphotograph, 50 µm.

Following a small PHA-L injection into area 9 (Fig. 5, case 133), a few neurons picked up the tracer, thereby allowing a complete chart of all labelled fibres in the striatum. The dense cluster of labelled fibres was located in the medial caudate n., consistent with the location of the focal projections seen in the other cases. In addition, there were isolated fibres in the lateral caudate n. and in the putamen (Fig. 5), consistent with the diffuse fibre projections from this area. These data demonstrate that a small confined injection site results in a focal projection terminating in a circumscribed region and a diffuse projection involving a larger striatal area.

#### Area 8A-FEF

**Fig. 6:**
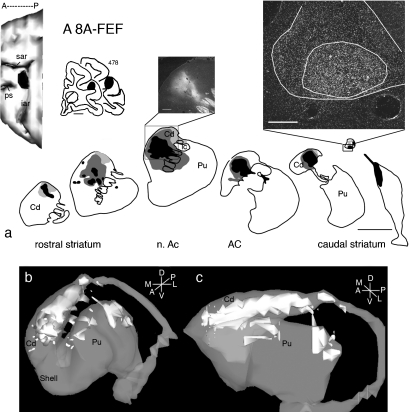
Projection from area 8A-FEF to the striatum. (a) Schematic chartings and microphotographs of the terminals following an injection into area 8A-FEF (case 478). The injection site in area 8A-FEF is illustrated through coronal brain sections and the relative 3-D reconstruction of the prearcuate region. The focal projection fields (black solid areas) and the diffuse projection fields are shown in charts at different AP levels: rostral striatum, AP 28–26; n. Ac, AP 22; AC, AP 18.5; and caudal striatum, AP 14–10. The diffuse terminal field for this tritiated AA injection is shown as a shaded area in grey surrounding the focal projections. illustrate the focal and diffuse terminal fields. b and c: 3-D rendering of the focal collective projections from the area 8A-FEF shown in coronal (b) and lateral (c) views of the striatum.Scale bars in (a), 5 mm (schematics); 1 mm and 200 µm (microphotographs at low and high magnifications, respectively).

There was one injection into area 8A. The injection site was located in the lateral bank of the superior arcuate sulcus and adjacent cortical convexity, caudal to the principal sulcus (Figs 2 and 6a). The injected region probably involved the FEF. However, we cannot rule out the possibility that the injection site included other functional parts of area 8A. This injection resulted in corticocortical labelling in lateral area 9, in rostral and caudal 46 and the SEF. In addition, a little labelling was found in PMdr but not in areas 24b and c.

#### Focal projections.

Fibres from area 8A-FEF entered at the rostral striatum, terminating throughout its rostrocaudal extent. In correspondence with, and rostral to, the level of the n. accumbens the focal projection field was primarily located in the central caudate n., involving the striatal region adjacent to the cell bridges of the internal capsule. There were also isolated patches of dense terminals in the medial and central putamen especially at the anterior commissure level. The labelling in the putamen was denser at very caudal levels, involving the most medial and dorsal aspect of this nucleus (Fig. 6a).

#### Diffuse projections.

The ability to detect the entire diffuse projection field of area 8A-FEF was limited by the nature of tritiated AA injections. Nonetheless, silver grain deposits were visible surrounding the focal projections and were particularly evident in the caudate n. and through the bridges of the internal capsule (Fig. 6a). Moreover, following this injection the diffuse projections occupied the dorsal caudate n., particularly at the n. accumbens level.

#### SEF

There were two injections into the SEF. The injection sites were located in the very rostral and dorsal area 6, close to the medial wall (Figs 2 and 7). We injected tritiated AA in one case (case 96) and FR in a second case (case 184). These injections resulted in corticocortical labelling primarily in area 8A-FEF and PMdr. In addition, there were weaker connections with areas 9, 46 and 24c.

#### Focal projections.

Fibres from the SEF entered the striatum posterior to those from area 8A-FEF and terminated in the lateral part of the caudate n. (Fig. 7). Unlike area 8A-FEF, the focal projection field did not extend to the rostral pole of the striatum. Furthermore, it terminated lateral to that from area 8A-FEF, within the internal capsule cell bridges and the putamen. In correspondence with, and posterior to, the level of the anterior commissure there were dense projections in the dorsal and medial putamen, as observed for area 8A-FEF (Fig. 7).

#### Diffuse projections.

Following the tritiated AA injection (Fig. 7a, case 96), silver grain deposits were visible surrounding the focal projections and were particularly evident in the caudate n. and through the bridges of the internal capsule, similar to the projection field from area 8A-FEF injection. In particular, the diffuse projection extended into the dorsal and medial caudate n., as confirmed by the FR injection into the SEF (Fig. 7b, case 184). In contrast to the tritiated AA labelling, the FR labelling allows the detection of the fibre morphology. Therefore, following this injection additional small clusters of terminals and single fibres were seen throughout the putamen (Fig. 7b).

#### PMdr

**Fig. 8:**
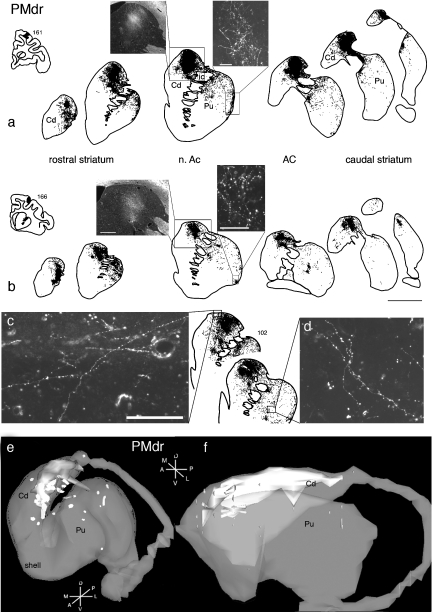
Projections from PMdr to the striatum. (a and b) Schematic chartings and microphotographs of the terminals following injections into PMdr (a, case 161; b, case 166). The focal projection fields (black solid areas) and the diffuse projection fields are shown in charts at different AP levels: rostral striatum, AP 28–26; n. Ac, AP 22; AC, AP 18.5; and caudal striatum, AP 14–10. Microphotographs at low and high magnifications illustrate the focal and diffuse terminal fields. (c and d) Microphotographs at high magnification illustrate axons with terminals representing some of the diffuse projections (case 102) extending in (c) the dorsomedial Cd n. and in (d) the putamen. (e and f) 3-D rendering of the focal collective projections from PMdr shown in (e) coronal and (f) lateral views of the striatum. Scale bars, 5 mm (schematics in a and b), 1 mm and 50 µm (low- and high-power, respectively, microphotographs in a and b); 100 µm (microphotographs in c and d).

There were four injections into PMdr. One injection (case 161, Fig. 8a) was placed at the same AP level but lateral to the SEF injection site. Following these injections in PMdr, the dense connections were with the other premotor areas. In addition, there were weaker connections with area 9 (primarily the lateral part), rostral and caudal area 46 and areas 24b and 24c whereas there was little, if any, labelling in area 8A-FEF.

#### Focal projections.

In contrast to the SEF projections, fibres from the PMdr entered at the rostral pole of the striatum. At the n. accumbens and anterior commissure levels, the focal projections from PMdr occupied the dorsal and lateral caudate n. The terminal fields also extended through the cell bridges and to the adjacent part of the putamen. At very caudal levels, these focal projections primarily occupied the dorsal caudate n., although there were also a few dense patches in the dorsal putamen (Fig. 8a and b).

#### Diffuse projections.

While the main focal projection field from the PMdr was located in the dorsal and lateral caudate n., the diffuse projections spread in the medial half of the caudate n. (Fig. 8a–c) and the dorsal and lateral putamen, rostral to and at the level of the anterior commissure (Fig. 8a, b and d). There were no labelled fibres in the ventral striatum. However, at these rostral levels there were clusters of fibres along the lateral border of the putamen (Fig. 8a and b). At more caudal levels, the diffuse projection field shifted its position from lateral to more medial regions of the dorsal putamen (Fig. 8a and b).

#### Areas 24c and 24b

**Fig. 9:**
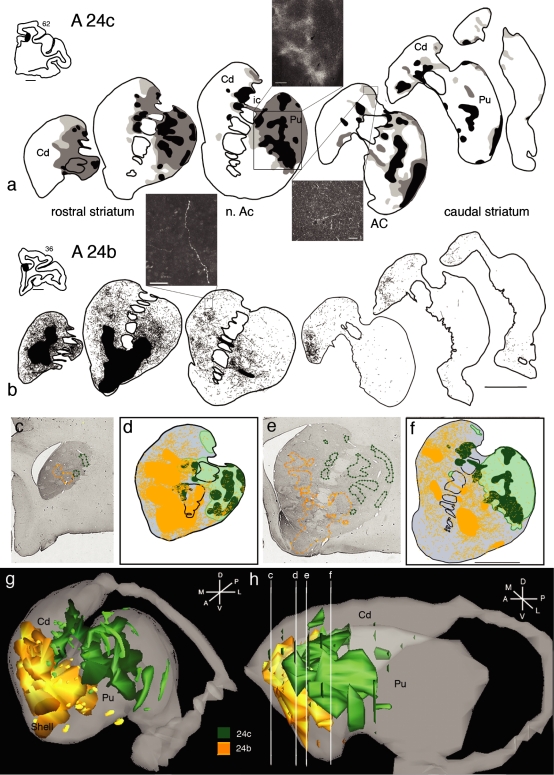
Projections from the dACC areas to the striatum. (a and b) Schematic chartings and microphotographs of the terminals following injections into area 24c (a, case 62) and area 24b (b, case 36). The focal projection fields (black solid areas) and the diffuse projection fields are shown in charts at different AP levels: rostral striatum, AP 28–26; n. Ac, AP 22; AC, AP 18.5; and caudal striatum, AP 14–10. The diffuse terminal field for the tritiated AA injection (a, case 62) is shown as a shaded area in grey surrounding the focal projections. Microphotographs at low and high magnification illustrate the focal and diffuse terminal fields. c - f: coronal slices through the 3-D model with the corresponding Nissl section. In d -f, the maps of the diffuse terminal fields from areas 24c and 24b are superimposed on the collective focal projections from these areas. Dark green, area 24c; orange, area 24b. g-h: 3-D rendering of the focal collective projections from the dorsal anterior cingulate areas shown in coronal (g) and lateral (h) views of the striatum. White lines in h indicate the level of sections illustrated in c-f. Scale bars, 5 mm (schematics in a and b), 1 mm and 50 µm (low- and high-power, respectively, microphotograph in a), 100 µm (microphotograph in b).

There were two injections into area 24c and three injections into area 24b. Injections into area 24c were located in the fundus of the cingulate sulcus at the anterior–posterior levels of the rostral premotor areas, just rostral to the genus of the arcuate sulcus (Figs 2 and 9a). These injections resulted in dense corticocortical labelling in the SEF and PMdr. In addition, there were weaker connections with the caudal premotor areas but not with area 8A-FEF. We compared corticostriatal projections from area 24c to those from area 24b (previously described in detail in Haber *et al*., 2006), whose injection sites were located rostral to those of 24c (Figs 2 and 9b) and resulted in corticocortical labelling primarily in areas 9 and 46, 24c, the ventral medial prefrontal cortex (vmPFC) and OFC.

#### Focal projections.

Unlike projections from other frontal regions, the focal projections from both areas 24c and 24b were less confined and extended throughout a larger striatal area. Nevertheless, there was a topographic organization between the terminal fields from these two regions of area 24 (Fig. 9). Following injections into area 24c there were several patches of dense terminals in the rostral striatum, primarily located in the putamen. These focal projections extended through the cell bridges of the internal capsule. At these levels, there were no focal terminal projections in the medial half of the caudate n. or in the ventral striatum (Fig. 9a and c–f). There was little convergence between the focal projections from 24c with those from 24b (Fig. 9b and c–f). At more caudal levels, patches of focal projections from 24c were more scattered and found throughout the striatum, with some located in the caudal ventral caudate n. and putamen. Taken together, confined small injections into different regions of area 24 resulted in focal projection fields that spread throughout a wide striatal region.

#### Diffuse projections.

Following injections into area 24c, silver grain deposits over background levels surrounded the focal terminal field extending the projection territory from area 24c throughout much of the rostral putamen and lateral caudate n. (Figs 1d and e, and 9a). However, these projections did not reach into the medial half of the striatum or into the territory of the ventral striatum. In contrast, diffuse projections from area 24b occupied a larger region of the rostral striatum including both the dorsal and ventral parts, invading the territories of all the other functional regions including those of 24c (Fig. 9a–f). Of particular interest is that the combined focal and diffuse projections from areas 24c and b occupied the entire rostral striatum with the exception of the shell region (Fig. 9d and f). Although the focal terminal field from area 24b primarily occupied rostral striatal regions, the diffuse projections from this area extended posterior to the anterior commisure level. At these levels, diffuse projections from 24b were primarily distributed in the medial caudate n. whereas focal and diffuse projections from area 24c were distributed in the lateral part of the caudate n. (Fig. 9a and b).

### Relationship between focal corticostriatal projections

**Fig. 10:**
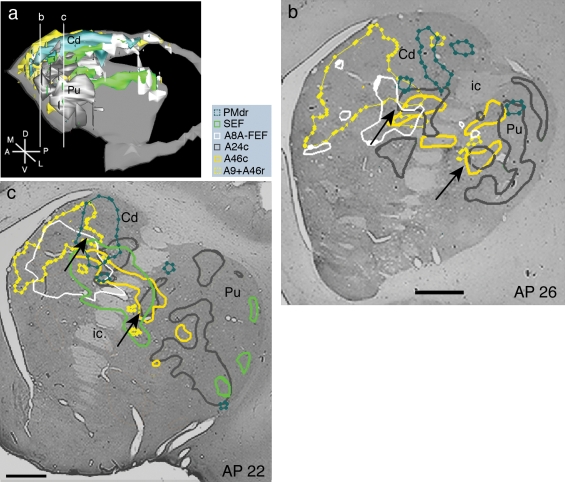
3-D rendering of the combined focal projections from all the frontal regions, illustrating the overlap between corticostriatal projections. The white lines in (a) the lateral view of the striatum indicate the level of sections illustrated in (b) and (c) (b and c) Coronal slices through the 3-D model with the corresponding Nissl section at the AP levels corresponding to (b) the rostral striatum (AP 26) and to (c) the n. Ac (AP 22). Note that the striatal sections shown in (b) and (c) have different magnifications. Black arrows indicate key striatal regions receiving convergent projections from areas 9 and 46, 8A-FEF, SEF, PMdr and 24c. Yellow dotted line, areas 9 and rostral 46; dark yellow, caudal area 46; white, area 8A-FEF; green, SEF; blue-green dotted line, PMdr; grey, 24c. Scale bars, 2 mm.

**Fig. 11:**
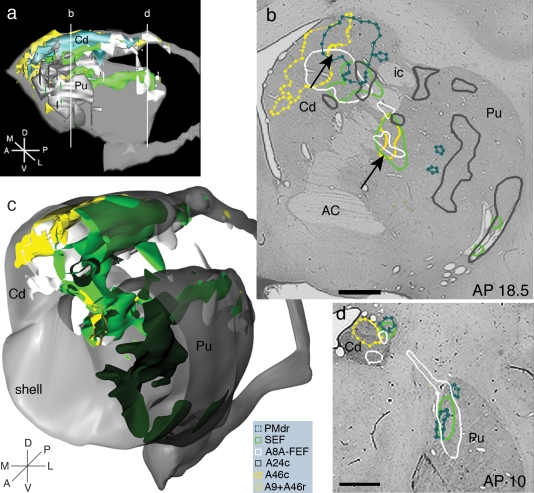
3-D rendering of the combined focal projections from all the frontal regions, illustrating the overlap between corticostriatal projections. The white lines in (a) the lateral view of the striatum indicate the level of sections illustrated in (b) and (d) (b and d) Coronal slices through the 3-D model with the corresponding Nissl section at the AP level corresponding to (b) the AC (AP 18.5) and to (d) the caudal striatum (AP 10). Black arrows indicate key striatal regions receiving convergent projections from areas 9 and 46, 8A-FEF, SEF, PMdr and 24c. Yellow dotted line, areas 9 and rostral 46; dark yellow, caudal area 46; white, area 8A-FEF; green, SEF; blue-green dotted line, PMdr; grey, 24c. (c) 3-D rendering of the striatum, clipped to illustrate the relationship between focal projections from each functional region. Scale bars, 2 mm.

The 3-D reconstruction of the collective focal projections and the dual cortical injections demonstrated both potential convergence and segregation in the striatum among the focal terminal fields arising both within and between different functional regions of cortex. Specific interface between cortical projections did not extend throughout the striatum but rather was specific to selective rostrocaudal levels (Figs 10 and 11). Moreover, within each rostrocaudal level, convergence between cortical projections was located in restricted regions.

#### Rostral striatum

At the most rostral striatal levels (Fig. 10b; AP 26), projections from areas 9 and 46 (yellow) and area 24c (grey) occupied a quite large portion of the dorsocentral striatum. In comparison, the area occupied by terminal fields from the area 8A-FEF (white) and PMdr (blue-green, dotted line) was somewhat smaller, and there were no focal projections from SEF (green). Projections from PMdr and areas 9 and 46 occupied primarily separated regions. In contrast, there was potential convergence between projections from areas 9 and 46, 8A-FEF and 24c. In particular, the 3-D reconstruction indicated that terminals from the caudal 46 (dark yellow) and 24c might converge in a central region of the striatum, adjacent to the internal capsule (Fig. 10b).

#### N accumbens level

**Fig. 13:**
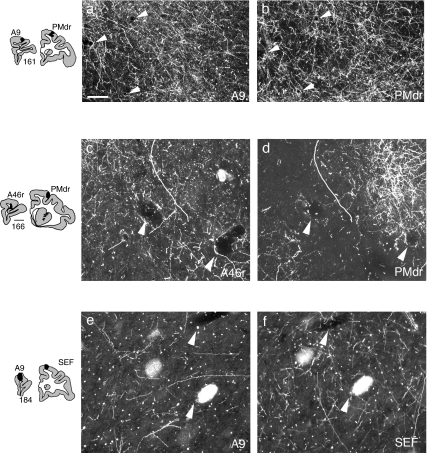
Microphotographs of adjacent coronal sections of the striatum showing the labelled terminals following dual tracer injections into different frontal areas. Labeled corticostriatal fibres following injections into (a) lateral area 9 and (b) PMdr in case 161, into (c) rostral area 46 and (d) PMdr in case 166, and into (e) areas 9 and (f) the SEF in case 184. White arrowheads indicate the corresponding blood vessels, and also the same white matter bundle in (e) and (f) in the adjacent sections. White lines in (c) and (d) indicate the border of the diffuse projections from PMdr. Note that dense clusters of fibres from (c) rostral area 46 and (d) PMdr primarily interdigitate but there is overlap between diffuse projections from these areas. There is also overlap between the diffuse projections from (e) area 9 and (f) those from the SEF. Scale bar, 100 µm (microphotographs), 5 mm (drawings illustrating the injection sites).

In contrast to more rostral levels, at the level of the n. accumbens there was convergence of focal projections from PMdr and area 9, particularly in the dorsal caudate n. (Fig. 10c; AP 22), as demonstrated by the dual cortical injections in case 161 (Fig. 13a and b). Moreover, based on the 3-D reconstructions, focal projections from caudal area 46 appeared to converge with those from areas 8A-FEF, SEF and 24c in another striatal region adjacent to the internal capsule, involving both the lateral caudate n. and the medial putamen (Fig. 10c).

#### Anterior commissure and caudal striatum levels

Consistent with that observed for the accumbens levels, at the anterior commissure level the dorsal striatum received convergent projections from area 9 and PMdr in the central caudate n. and from caudal area 46 and areas 8A-FEF, SEF and 24c in the region adjacent to the internal capsule involving both the lateral caudate n. the medial putamen (Fig. 11b and c; AP 18.5). There was also segregation of the terminal fields. For example, focal projections from area 24c spread to the central and ventral part of the putamen, which did not receive focal projections from the other cortical regions (Fig. 11b). At caudal striatal levels (Fig. 11d; AP 10) there was little or no convergence between focal projections from areas 9 and 46 and PMdr. In fact, terminals from these cortical regions were segregated, occupying somewhat different but adjacent locations in the caudate n. In contrast, potential convergence between focal projections from area 8A-FEF and the SEF was still present in the dorsomedial putamen (Fig. 11d).

### Overlap of diffuse and focal projections

**Fig. 12:**
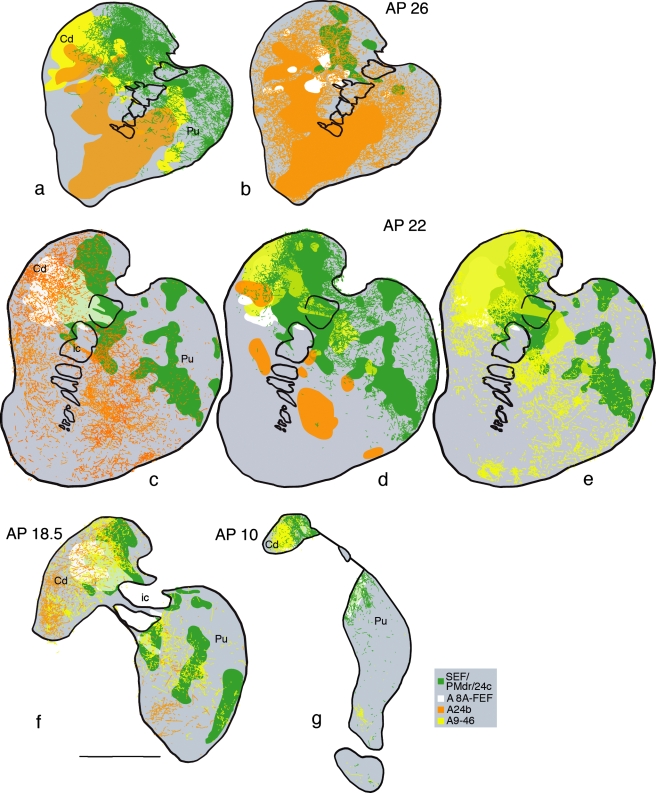
Chartings of the terminal fields from each cortical functional region, illustrating the collective diffuse projections superimposed on the focal terminal fields at four chosen AP levels: (a and b) rostral striatum (AP 26); (c–e) n. Ac (AP 22); (f) AC (AP 18.5); and (g) caudal striatum (AP 10). Yellow, areas 9 and 46; orange, area 24b; white, area 8A-FEF; green, SEF, PMdr and 24c. In (a) and (d), orange is transparent to better illustrate the convergence between focal projections from area 24b with those from areas 9 and 46; in (d)– (e), yellow is transparent to better illustrate the convergence between focal projections from areas 9 and 46 with those from areas 8A-FEF, SEF, PMdr and 24c (the convergence area appears in green); in (c), (f) and (g), white is transparent. Note that diffuse projections from each cortical region distribute to a larger striatal area, extending the territory covered by the focal projections. Scale bar, 5 mm.

The diffuse projections extended from each focal projection, widening the striatal area that received input from each cortical domain. This diffuse projection system amplifies the potential interface between corticostriatal terminals in the striatum. Figure 12 illustrates the combined diffuse projection from each cortical area. The combination of focal and diffuse projections from the collective injection sites spread throughout the entire rostral striatum from the rostral pole to the anterior commissure (Fig. 12a–f; AP 26–18.5) with the possible exception of parts of the shell region. However, at the level at which the internal pallidal segment is fully developed (AP 14.5), much of the putamen did not receive projections from any of these frontal areas.

Diffuse projections from PMdr and SEF were distributed throughout the dorsal and central part of the rostral striatum interfacing with focal projections from areas 9, 46 and 24b (Fig. 12a). This was unlike that observed for the focal projections from PMdr, SEF and 24c and those from areas 9, 46 and 24b that scarcely interfaced at the rostral striatal levels. Similarly, diffuse projections from 24b invaded the terminal fields from PMdr, SEF and 24c in the dorsal striatum, although there was little convergence between the focal projections from these areas (Fig. 12b). This was also observed posteriorly, at the levels of the n. accumbens and anterior commissure, where diffuse projections from 24b spread in the striatal territory receiving focal projections from PMdr, SEF and 24c, along with those from 8A-FEF (Fig. 12c). In contrast, diffuse projections from PMdr and SEF did not spread in the ventral striatum and therefore there was almost no overlap with the focal projections from 24b (Fig. 12d). However, diffuse projections from PMdr and SEF widely spread throughout the dorsal caudate n. overlapping with focal projections from areas 9 and 46 (Fig. 12d). In a ‘complementary’ way, diffuse projections from areas 9 and 46 extended laterally in the caudate n. and putamen, overlapping with focal projections from PMdr, SEF and 24c (Fig. 12e and f). While there was some potential convergence between the diffuse projections from areas 9 and 46 and the focal projections from PMdr, SEF and 24c at more caudal striatal levels, it was very limited (Fig. 12g).

In summary, the model predicted that focal projections from area 9 and PMdr would converge in the dorsal caudate n. at the n. accumbens and anterior commissure levels. The model predicted also that, at the same anterior–posterior levels, there would be convergence between focal projections from caudal area 46, 8A-FEF, SEF and 24c in a region of the medial putamen, adjacent to the internal capsule. Moreover, it also showed that there would be little or no convergence between focal projections from PMdr and rostral area 46, or between focal projections from area 9 and the SEF. However, there could be a potential interaction between diffuse and focal projections from these cortical areas. In order to test these striatal regions of potential convergence we carried out dual cortical injections in three cases. The results showed that there was convergence of the focal projections from area 9 and PMdr in the dorsal caudate n. (case 161). Figure 13a and b, case 161, illustrates dense fibre clusters from area 9 and PMdr located in the same striatal area of the mediodorsal caudate n. In addition the model predicted that, in the same region of the caudate n., convergence between focal projections from PMdr and rostral area 46, and between focal projections from area 9 and the SEF, would be limited. This was demonstrated in two cases. Case 166 had one injection into rostral area 46 and one into PMdr. Case 184 had one injection into area 9 and one into the SEF. Both of these two cases confirmed that the focal projections did not converge. However, there was convergence between diffuse projections from these cortical areas (Fig. 13c and d, case 166; Fig. 13e and f, case 184). Thus, the 3-D reconstruction and the dual cortical injections indicated that two regions of the dorsal caudate n. and medial putamen, at the level of the n. accumbens, were sites of convergence between focal and diffuse projections from these different frontal areas. To further confirm these relationships, we selectively placed two injections of retrogradely transported tracers into these regions of the dorsal striatum.

### Retrograde tracer injections into the convergence regions of the striatum

**Fig. 14:**
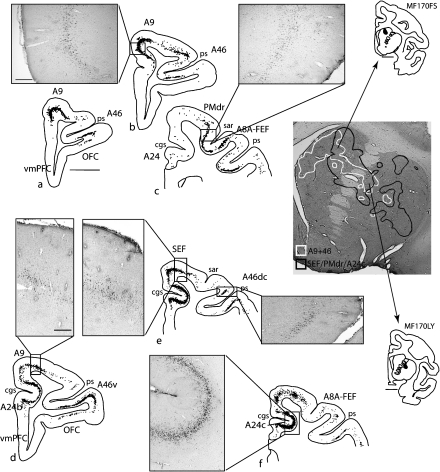
Drawings and microphotographs of the retrogradely labelled neurons following two tracer injections into the striatum at the level of the n. Ac, in the dorsal and medial Cd n. (case 170, FS) and in the medial putamen (case 170, LY), that are sites of potential convergence between projections from areas 9 and 46 (white) and SEF, PMdr and 24c (black) as predicted by the 3-D model and by the dual cortical injections. a - f: coronal sections of the brain showing the labelled corticostriatal neurons in the relevant frontal areas at different AP levels. Microphotographs show the labelled neurons in (b) medial area 9 and (c) PMdr following injection into the dorsomedial Cd n. (case 170, FS) and the labelled neurons in (d) area 9, in (e) the SEF and in dorsal and caudal area 46, and in (f) area 24c following injection into the medial putamen (case 170, LY). Scale bars, 5 mm (drawings), 500 µm (microphotographs).

We placed one injection into the dorsal caudate n., at the n. accumbens level (Fig. 14, case 170, FS), to confirm the convergence between corticostriatal terminals from areas 9, 8A-FEF and PMdr, as predicted by the 3-D reconstruction. Following this injection, retrogradely labelled neurons were primarily distributed in areas 9 and 46 and, as expected, in PMdr and area 8A-FEF (Fig. 14a–c). Additionally, there were also labelled cells (albeit fewer) in areas 24b and 24c, confirming that the dorsal caudate n. received also diffuse projection from the dACC. As predicted, there were no labelled cells in the vmPFC (Fig. 14a–c). Finally, we placed a second injection into the medial putamen, at the n. accumbens level (Fig. 14, case 170, LY), to confirm the convergence between corticostriatal terminals from area 46, SEF and area 24c which was predicted by the 3-D model. Following this injection, there were retrogradely labelled neurons primarily in areas 9 and 46 and, as expected, in area 24c and the SEF (Fig. 14d–f). In addition, there were also retrogradely labelled neurons in area 24b and PMdr, confirming that diffuse projections from area 24b and isolated dense clusters of terminals from PMdr terminate in the dorsomedial putamen (Fig. 14d–f). These results are in agreement with our previous findings showing that following tracer injections into restricted striatal locations there were retrogradely labelled cells in multiple cortical functional regions (Haber *et al*., 2000; McFarland & Haber, 2000).

## Discussion

Our results reveal some important features of the corticostriatal projections from areas 9 and 46 and areas 8A-FEF, SEF, PMdr and 24c. (i) Focal projections from different sectors of areas 9 and 46 form a complex topography which may reflect functional diversity within this cortical region. (ii) A diffuse projection system appears to be a common feature of the corticostriatal projections from areas 9, 46 and 24b and those from areas 8A-FEF, SEF, PMdr and 24c, and may help to ‘broadcast’ the cortical activity from a specific cortical region to a larger striatal area. (iii) There is convergence between projections from areas 9 and 46 with those from areas 8A-FEF, SEF, PMdr and 24c in selective striatal regions. (iv) Focal projections from areas 24c and 24b of the dACC, which define two separate striatal territories, both have widespread terminal fields. This characteristic places the corticostriatal terminals from the dACC in a unique position to interact with terminals from several frontal areas.

### Technical considerations

All of our injection sites were relatively small and confined to specific frontal regions. As shown in Fig. 2, our injection sites did not cover an entire cortical region. This approach was necessary to demonstrate the topographic organization of projections from different cortical areas as well as from within each cortical area. For example, we could demonstrate the variation of focal projections from within regions of areas 9 and 46. Our 3-D renderings were based on the focal projections from the collective individual injection sites. However, as our results do not include the entire striatal territory devoted to each cortical area, they are not likely to demonstrate the complete projection field. Thus, the areas of potential convergence between corticostriatal terminal fields may be an underestimate given that some cortical regions may have a larger focal projection field. To verify the potential convergence as predicted by the 3-D model, we injected two cortical areas in the same animal (three cases). In addition, we selectively placed two injections of retrogradely transported tracers into the striatal regions of potential convergence.

Convergence between focal projections refers to the striatal area that is potentially occupied by more than one terminal field. We do not mean to imply a synaptic association, which would require electron microscopy (EM). However, previous studies in primates have shown, at the light-microscopic level, that terminals from functionally related sensorimotor cortical regions converge in the striatum (Flaherty & Graybiel, 1991), and EM studies in rodents have shown that sensory and motor inputs terminate on the same neuron (Ramanathan *et al*., 2002).

### Topography of the focal projections from areas 9 and 46

Projections from different parts of areas 9 and 46 (Walker, 1940; Barbas, 1992; Petrides & Pandya, 1999) both terminate in a topographic manner but also converge in selective striatal levels. We previously showed that focal projections from different parts of a cortical functional region, such as those from the vmPFC or area 24b, terminate in the same striatal area (Haber *et al*., 2006). However, we also showed that projections from different areas of the OFC did have a topographic organization similar to those from areas 9 and 46.

The topographic organization of corticostriatal projections is, in general, considered to be related to different functional domains of the cortex (Middleton & Strick, 2002). Therefore, the fact that regions within areas 9 and 46 terminate within the dorsal striatum in somewhat different locations suggest differential functional properties associated with these prefrontal areas. However, associating different area 9 and 46 sectors with specific functions remains elusive. Moreover, the majority of studies in awake, behaving nonhuman primates have focused on area 46, with few in area 9 (Funahashi *et al*., 1993; Constantinidis *et al*., 2001; Wallis *et al*., 2001). Functional variations between areas 9 and 46 are supported by lesion studies (for a review, see Petrides, 2000) and by differences in their corticocortical connectivity (Barbas, 1988; Petrides & Pandya, 1999). Our data from the injections into area 9 suggest potential differences between the medial and lateral parts of this area. Lateral area 9 is cortically connected to areas 46, 8A and PMdr; in contrast, medial area 9 is primarily connected with the anterior cingulate areas 32 and 24b. Moreover, the cortical projections from medial and lateral area 9 terminate in different locations of the rostral striatum. Potential functional differences between medial and lateral parts of area 9 are also supported by studies in humans. Recent imaging data suggest that the medial aspect of the prefrontal cortex, involving medial area 9, may be associated with perception and inference of mental state (Frith & Frith, 2006; Mitchell *et al*., 2006).

### Diffuse projections

There is a diffuse projection system from areas 8A-FEF, PMdr, SEF and 24c, sending fibre clusters throughout a wide striatal area. This is consistent with our previous reports showing a diffuse projection from all prefrontal areas (including OFC and vmPFC; Haber *et al*., 2006). Diffuse projections from PMdr terminated throughout the striatum, but not in its ventromedial part. In contrast, these projections extended into the dorsomedial caudate n., the territory that receives focal projections from area 9, and into the region of the putamen that receives focal projections from the SEF and area 24c. Taken together, these results suggest that the diffuse projections enlarge the terminal field from each cortical region, extending the potential integrative network. Likewise, the diffuse projection from area 24b innervated striatal terminal fields from areas 8A-FEF, SEF, PMdr and 24c.

The study of corticostriatal projections has primarily concentrated on the focal projections due to the relatively large, coordinated input required to activate the medium spiny neurons (Wilson, 2004). However, a diffuse projection system appears to be a general rule of corticostriatal projections and is likely to serve an important, but perhaps separate, function from that of the focal projections. In general, this innervation might be activated at key times to ‘publicize’ specific cortical activity that sets the tone by which other information (via focal projections) is received. Thus, under specific environmental conditions, activation of the diffuse projection system may prime the striatum to favour activation from other corticostriatal pathways. For example, the diffuse fibres from area 24b that reach the SEF, PMdr and 24c terminal fields may be critical for alerting a wider striatal region about the general internal state of the animal for future action. As previously proposed, this activation may occur through targeting a separate set of neurons or receptors than those activated by the focal projections (Haber *et al*., 2006).

### Relationship between focal corticostriatal projections

Our results indicate that there is some convergence between terminals from different functional cortical regions. This is consistent with previous data that show convergence among pathways within a specific function (Flaherty & Graybiel, 1991; Parthasarathy *et al*., 1992) or between them (Haber *et al*., 2006). Yeterian & Pandya (1991) showed that there is convergence between terminals from cortical areas that are strongly corticocortically connected, and suggested that this anatomical organization could be a feature of the corticostriatal pathways. In contrast, Selemon & Goldman-Rakic (1985) showed primarily interdigitation between corticostriatal projections from areas which are cortically connected. Our results are in agreement with both organizational schemes (convergence and interdigitation). In addition, our results showed that, in two key regions, there is also potential convergence between corticostriatal terminals from areas that do not have strong cortical connections. One region adjacent to and surrounding the internal capsule involved the lateral caudate n. and the medial putamen. The second region was located in the dorsal caudate n. These data suggest that the striatum not only processes information through functionally discrete circuits but also is in position to integrate information between cortical functional domains. Interestingly, these integrative regions are selective, and may be the key areas for a neural network necessary for adaptive actions. Indeed, flexible change in the cortical striatal activity is a crucial element in learning, and the striatum may be the optimal site for integrating information from several cortical regions, thus reducing the computational demands for learning (Toni *et al*., 2002).

The region adjacent to the internal capsule received projections from the SEF and area 24c. The SEF signals error in saccade preparation in experimental conditions in which a planned saccade is withheld (Stuphorn *et al*., 2000). In contrast, area 24c neurons code the reward occurrences at different times of this task and may signal comparison between the predicted and actual action (Ito *et al*., 2003). This striatal region also received projections from area 8A-FEF. This injection probably included both the FEF, which is associated with saccadic eye movements, and other area 8A regions which are involved in mnemonic and oculomotor processing but not specifically with saccades (Robinson & Fuchs, 1969; Bruce *et al*., 1985; MacAvoy *et al*., 1991; Rowe & Passingham, 2001). In addition, this striatal region adjacent to the internal capsule received a focal projection from caudal area 46. Convergence between projections from areas 46 and 8A is not surprising given that both these areas are involved in working memory processing (Funahashi *et al*., 1989), albeit in different aspects (Rowe *et al*., 2000). Functional imaging studies in humans show that area 46 is particularly important for executive processing of working memory (selection of information from memory), whereas area 8A is involved in on-line maintenance of information during a delay period (Petrides *et al*., 1993; D'Esposito *et al*., 1998; Miller, 1999; Rowe *et al*., 2000; Rowe & Passingham, 2001; Lebedev *et al*., 2004).

A second striatal region of the dorsal and medial caudate n. received convergent projections from area 9 and PMdr. The PMdr is conventionally grouped with other premotor regions, based on functional studies and its strong connections with caudal premotor areas (Rizzolatti *et al*., 1998; Barbas & Pandya, 1987; di Pellegrino & Wise, 1993; Rizzolatti & Luppino, 2001; Simon *et al*., 2002). However, recently an argument has been made that the PMdr may, from a functional prospective, be more like prefrontal areas than motor areas (Boussaoud, 2001; Picard & Strick, 2001). If PMdr is classified as more prefrontal-like, then convergence of fibres from these areas indicates interaction between regions processing different aspects of executive function. However, if PMdr is considered premotor than convergence of terminals with area 9 in the striatum suggests potential interaction between prefrontal and motor-control areas.

### Areas 9 and 46 are in a pivotal position

Of particular interest is that convergence between focal projections from areas 9 and 46 with those from areas 8A-FEF, SEF, PMdr and 24c takes place in specific and selected striatal regions, at levels just rostral to and at the anterior commissure. At more anterior levels, these projections remain relatively segregated. However, there is convergence at these rostral levels between focal projections from areas 9 and 46 and those from the OFC and 24b/32, areas that are more closely associated with reward value, risk assessment and motivation (Petrides *et al*., 2002; O'Doherty *et al*., 2003; Wallis & Miller, 2003; Roesch & Olson, 2004; Haber *et al*., 2006). Areas 9 and 46 are in a pivotal position in the distributed frontal network of cognition and learning, with functional and connectional interfaces with the anterior cingulate cortex, OFC and premotor areas. Taken together, the projections from areas 9 and 46 appear to have a similar position in the striatum, converging at rostral levels with inputs from areas associated with motivation and reward value (rostral striatum) and at more posterior levels (n. accumbens and/or anterior commissure levels) with those from cortical areas associated with action planning. These potential interactions between cortical regions may serve a different function in the striatum. The striatum receives focal cortical projections that are funnelled into a concentrated region, along with an input from the dopamine cells that signal reward and/or salience (Schultz, 2002; Redgrave & Gurney, 2006). Moreover, there is a unique relationship between these inputs on to the medium spiny neurons (Bouyer *et al*., 1984). Thus, the pivotal role of areas 9 and 46 in the striatum may differ from its role in cortex due to the combination of concentrated focal projections and the unique dopaminergic input, thereby placing it in a position to specifically mediate learning and the development of routines.

### Widespread dACC terminal fields

Focal terminal fields from areas 24c and 24b defined distinct striatal territories. Terminals from area 24c occupied much of the putamen, which also receives other motor-related inputs, consistent with the role of this area in motor control (Luppino *et al*., 1991; Takada *et al*., 1998; Nambu *et al*., 2002). In contrast, projections from area 24b involved much of the rostral and ventral caudate n. and putamen and converged with those from the OFC and areas 9 and 46 in the rostral striatum, suggesting an important role for this striatal area for incentive-based learning (Haber *et al*., 2006). Despite their functional and connectivity differences, terminals from both 24c and 24b were distributed through a remarkably wide region of the striatum. This differs from most cortical projections, which terminate in more circumscribed parts of the striatum. The role of the dACC is monitoring actions in conflict situations. This cortical region is a unique part of frontal cortex, in that it contains within in it a representation of many diverse frontal lobe functions, including motivation, cognition and motor control, that are crucially important for monitoring (Paus, 2001). This role of the dACC may be reflected in its widespread terminations that ‘broadcast’ information over a large striatal area and therefore signal environmental variations to update basal ganglia-mediated routines.

### Functional considerations

The striatum is considered a fundamental component for learning, execution of routine behaviour, and adaptive habit expression (Hikosaka *et al*., 1999; Jog *et al*., 1999; Graybiel, 2005;Monchi *et al*., 2006). However, the role of the striatum in guiding and changing behaviours is complex. In some instances the striatum is the ‘teacher’ of the cortex (Pasupathy & Miller, 2005) but, in other experimental paradigms, learning occurs simultaneously in cortex and striatum (Brasted & Wise, 2004). Human imaging studies show that prefrontal and rostral premotor areas and the rostral striatum are activated preferentially at early stages of learning (Toni *et al*., 2001; Lehericy *et al*., 2005). However, there is variation in cortical vs. striatal temporal firing patterns in different stages of well-learned routine tasks (Fujii & Graybiel, 2005). Our results show that each cortical region sends a focal projection to the striatum, a part of this projection converging with focal projections from other functional domains. This finding indicates that the link between areas 9 and 46 and areas 8A-FEF, SEF, PMdr and 24c may occur not only in the cortex but also in the striatum, providing an anatomical substrate for the synchronized activation during learning tasks. This organizational scheme suggests anatomical substrates for both the parallel processing to carry out well-learned routines and the integrative capability of the striatum to help guide changes in behaviour.

## Abbreviations

A24b: area 24b
A24c: area 24c
A46: area 46
A46c: caudal area 46
A46dc: dorsal and caudal area 46
A46r: rostral area 46
A46v: ventral area 46
A6: area 6
A8A: area 8A
A9: area 9
A9l: lateral area 9
A9m: medial area 9
AA: amino acid(s)
AC: anterior commissure
AP: anterior–posterior level
Cd: caudate
cgs: cingulate sulcus
cs: central sulcus
dACC: dorsal anterior cingulate cortex
FEF: frontal eye field
FR: fluororuby
FS: fluorescein
iar: inferior arcuate sulcus
ic: internal capsule
ip: intraparietal sulcus
l: lateral sulcus
LY: lucifer yellow
n. Ac: nucleus accumbens
n.: nucleus
NGS: normal goat serum
OFC: orbital frontal cortex
PB: phosphate buffer
PHA-L: *phaseolus vulgaris*–leucoagglutinin
PMdr: dorsal and rostral premotor cortex
PMvr: ventral and rostral premotor cortex
ps: principal sulcus
Pu: putamen
sar: superior arcuate sulcus
SEF: supplementary eye field
sts: superior temporal sulcus
vmPFC: ventral medial prefrontal cortex.
